# HSP70 Gene Family in *Brassica rapa*: Genome-Wide Identification, Characterization, and Expression Patterns in Response to Heat and Cold Stress

**DOI:** 10.3390/cells11152316

**Published:** 2022-07-27

**Authors:** Javaria Tabusam, Qiling Shi, Daling Feng, Sumer Zulfiqar, Shuxing Shen, Wei Ma, Jianjun Zhao

**Affiliations:** State Key Laboratory of North China Crop Improvement and Regulation, Key Laboratory of Vegetable Germplasm Innovation and Utilization of Hebei, Collaborative Innovation Center of Vegetable Industry in Hebei, College of Horticulture, Hebei Agricultural University, Baoding 071000, China; javariatabusam77@gmail.com (J.T.); shiqiling6876@aliyun.com (Q.S.); fdlsm@hebau.edu.cn (D.F.); sumerzulfiqar408@gmail.com (S.Z.); shensx@hebau.edu.cn (S.S.)

**Keywords:** *Brassica rapa*, genome-wide, HSP70 family genes, expression profiling

## Abstract

Heat shock proteins protect plants from abiotic stress, such as salt, drought, heat, and cold stress. HSP70 is one of the major members of the heat shock protein family. To explore the mechanism of HSP70 in *Brassica rapa*, we identified 28 putative HSP70 gene family members using state-of-the-art bioinformatics-based tools and methods. Based on chromosomal mapping, HSP70 genes were the most differentially distributed on chromosome A03 and the least distributed on chromosome A05. Ka/Ks analysis revealed that *B. rapa* evolution was subjected to intense purifying selection of the HSP70 gene family. RNA-sequencing data and expression profiling showed that heat and cold stress induced HSP70 genes. The qRT-PCR results verified that the HSP70 genes in Chinese cabbage (*Brassica rapa* ssp. *pekinensis*) are stress-inducible under both cold and heat stress. The upregulated expression pattern of these genes indicated the potential of HSP70 to mitigate environmental stress. These findings further explain the molecular mechanism underlying the responses of HSP70 to heat and cold stress.

## 1. Introduction

The heat shock transcription factor family strengthens plants under biotic and abiotic stress conditions; as a result, plants proceed with normal growth and development [1]. Temperature significantly affects plant growth; all plants require an optimum temperature for survival. Thus, many studies have been conducted on plants to explore the response to temperature stress [2,3]. (Heat shock treatment has been shown to stimulate a genetic network of proteins as a defense system in plants, and these proteins are named heat shock proteins (HSPs) [4]. HSPs are divided into subgroups on the basis of their molecular weight (60, 70, 90, and 100 KDa), named HSP60, HSP70, HSP90, and HSP100 gene families, respectively [5]. Of these heat shock families, HSP70 has been identified as the most conserved throughout evolution and play vital role in the maintenance of normal plant development under heat stress that can be induced by exposure to sublethal temperatures in eukaryotes [6,7,8]. HSP70 act as chaperons to protect the plant under temperature stress condition by covering the heat and cold sensitive molecules. It helps to reform their folding structure and repairing the deformed structures. All the HSP70 proteins consists of two functional domains named as nucleotide binding domain (NBD) and substrate binding domain (SBD) [9].

The HSP70 family, which encodes HSP70 proteins, has been identified and studied in many plants, such as Arabidopsis (17 genes), rice (26 genes), spinach (12 genes), and soybean (61 genes) [8]. In *Arabidopsis*, HSP70 deficient plants showed the stunted plant growth and abnormal leaf phenotype [10], double HSP70 knockout leads to the defective male and female gametes [11]. Furthermore, the cytosolic and nuclear HSP70 proteins showed significant role in *Arabidopsis* plant development under abiotic stress conditions [12]. To date, little information of HSP70 genes has been elucidated in Brassica crops. In the current study, the HSP70 gene family will be explored in a *Brassica rapa* family member. This vegetable-based family includes cabbage, Chinese cabbage, mustard, and turnip, which are sources of oil, amino acids, and proteins for humans and animals. These vegetables are highly susceptible to temperature stress [13] and are adversely affected by heat, cool, drought, and biotic stress during their cultivation, which causes germination delays, growth arrest, frost damage, and poor head yield [14].

*Brassica rapa* has gained remarkable economic and nutritional value due to its heading trait, which makes it highly susceptible to stress. Under temperature stress, the leaves become pale and weakened due to chloroplast damage, and the plant becomes unable to properly regulate photosynthesis [15]. Under such conditions, the HSP70 gene family plays an important role in protecting the plant and reestablishing the cellular and biological functioning of plants. Genome-wide identification and characterization of the HSP70 gene family have not been studied in *Brassica rapa* to date. We identified and explored the members of the HSP70 gene family, constructed their evolutionary tree and synteny correlations, and predicted protein structures with available bioinformatics software. Moreover, we analyzed the expression of these proteins by qRT-PCR to determine their responses to heat shock stress in Chinese cabbage (*Brassica rapa* subsp. *Pekinensis*). These findings will open up new gates for understanding the structure, function, and expression of HSP70 genes in *B. rapa*.

## 2. Methodology

### 2.1. Identification and Characterization of the HSP70 Gene Family

In the first step of genome wide analysis of the HSP70 gene family to identify and characterization, the amino acid sequences of *A. thaliana* HSP70 family genes (AT3G12580.1) was obtained from the TAIR Arabidopsis genome database (TAIR-Home Page arabidopsis.org (accessed on 20 April 2022). This sequence was used as a query to retrieve the HSP70 genes in the Brassica database (http://brassicadb.cn/#/BLASTP/, accessed on 20 April 2022). Secondly, the HMMER 3.1 (http://www.hmmer.org, accessed on 20 April 2022) and BLASTP with the threshold e-value set to 1e^−^^5^ were performed using the Hidden Markov Model (HMM V 3.0) profiles of the HSP70 gene family (**PF00012**) were downloaded from the Pfam protein database (http://pfam.xfam.org/, accessed on 20 April 2022) and used as the inquiry, The default limitation of HMMER 3.1 was set to 0.01. Finally, total of 28 HSP70 were retrieved in the *Brassica rapa* genome. *B.rapaHSP70* proteins were further characterized by determining the molecular weight, number of amino acids, isoelectric point, and grand average of hydropathicity (GRAVY) through the ProtParam tool (ExPASy-ProtParam tool, accessed on 20 April 2022). The protein sequences were uploaded to the online database Wolf PSORT for the prediction of subcellular localization (https://wolfpsort.hgc.jp/, accessed on 20 April 2022). Furthermore, the protein conserved domain was identified using the NCBI conserved domain online server (www.ncbi.nlm.nih.gov, accessed on 20 April 2022) and motif analysis was performed by using MEME Suite (meme-suite.org, accessed on 20 April 2022). The gene structure was predicted by the Gene Structure Display Server (GSDS) (http://gsds.cbi.pku.edu.cn/, accessed on 20 April 2022), using the CDS and genomic sequences of 28 selected *B.rapaHSP70*. Furthermore, prediction of the subcellular location pattern of each *B.rapaHSP70* was carried out using the WoLF PSORT server (https://wolfpsort.hgc.jp/, accessed on 20 April 2022; [16]).

### 2.2. Phylogenetic Tree and Synteny Analysis of B.rapaHSP70 Family Proteins

The protein sequences of HSP70s from *Arabidopsis*, *B. oleracea*, and *B. napus* were retrieved to construct the phylogenetic tree using MEGA X (V 6.06) software (https://www.megasoftware.net/, https://wolfpsort.hgc.jp/, accessed on 20 April 2022). The sequences were multiple aligned and employed using the neighbor-joining (NJ) method with 1000 bootstrap replicates [17]. Synteny relationships of *B. rapa* with *B. oleracea*, *B. napus*, and *Arabidopsis* were developed using MCScanX to obtain collinearity files that were used to build dual synteny plots in TBtools (https://github.com/CJ-Chen/TBtools, accessed on 20 April 2022).

### 2.3. B.rapaHSP70 Duplicated Gene Identification and Purity Selection Analysis

Duplicated genes were retrieved using the Blast, MCScanX, and Advance Circos features of TBtools [18]. For the purity selection pressure of duplicated genes, the Ks/Ka value was calculated using the Ka/Ks Calculator in TbTools, and TMY was calculated using previous studies in *B. rapa* [19,20]. Synteny analysis was performed for the correlation prediction among *B. rapa* and its closely related species through TBtools [21].

### 2.4. Cis-Element Analysis of B.rapaHSP70 Gene Promoters and Protein Structure

Putative cis-elements of the 28 *B.rapaHSP70* genes were classified, and the 2000 bp upstream of the start codon were downloaded from the BRAD database (http://brassicadb.cn, accessed on 20 April 2022). The PlantCARE web-based tool (http://bioinformatics.psb.ugent.be/webtools/plantcare/html/, accessed on 20 April 2022) was used for further classification, and the results were presented using TBtools. The graphical construction of the 3-dimensional (3D) structure of *B.rapaHSP70* proteins was done using the online web-based software PHYRE2 (PHYRE2 Protein Fold Recognition Server (ic.ac.uk), selecting the fold recognition method [22].

### 2.5. Targeted miRNA Prediction and Functional Analysis

CDS sequences of *B.rapaHSP70* were used to determine the interaction of genes with microRNAs using the psRNATarget database (http://plantgrn.noble.org/psRNATarget, accessed on 20 April 2022), and the interactions were further prophesied by Cytoscape Software. Gene ontology (GO) annotation was performed to explore functional characteristics. The *B.rapaHSP70* gene sequences were uploaded to the online eggNOG database (http://eggnog-mapper.embl.de/, accessed on 20 April 2022). Then, the GO annotation data were handled by using (http://brassicadb.cn, accessed on 20 April 2022) and graphical demonstration was given by using OmicShare (https://www.omicshare.com/, accessed on 20 April 2022).

### 2.6. Digital Expression Analysis of B.rapaHSP70

RNA-seq data were used to determine the expression of *B.rapaHSP70* genes in different tissues, and a heat map was constructed using TbTools [23]. The data used for expression profiling were retrieved from the NCBI Gene Expression Omnibus (http://www.ncbi.nlm.nih.gov/geo/, accessed on 20 April 2022) under accession no. **GSE43245**. In addition, expression profiles of *B.rapaHSP70* under cold and heat stress conditions were obtained by performing qRT-PCR.

### 2.7. Plant Material and Stress Conditions

Seeds of Chinese cabbage were grown in pots containing soil: vermiculite mixture (3:1) in a controlled chamber (24/18 °C, 60–65% relative humidity, 16/8 day/night) until they reached the five-leaf stage. Cold and heat stress conditions were applied to five-leaf seedlings. Seedlings were transferred to 4 °C for cold stress and 45 °C for heat stress under the same day/night and humidity conditions. Three biological replications were used in both stress conditions. Leaf samples were harvested after 0, 1, 3, 6, 12, and 24 h, placed in liquid nitrogen, and stored at −80 °C [24].

### 2.8. RNA Extraction and qRT-PCR Analysis

Leaf samples were used to extract the RNA, and qRT-PCR was performed with three replicates to check *B.rapaHSP70* gene expression; actin was used as the internal reference gene in Chinese cabbage. All primers used in this experiment are listed in Table S4 The 2^−ΔΔCt^ method was used to check the relative expression levels, and the results were graphically represented [25].

## 3. Results

### 3.1. Identification and Characteristics of HSP70 Gene Family Members in B. rapa

**Figure 1:**
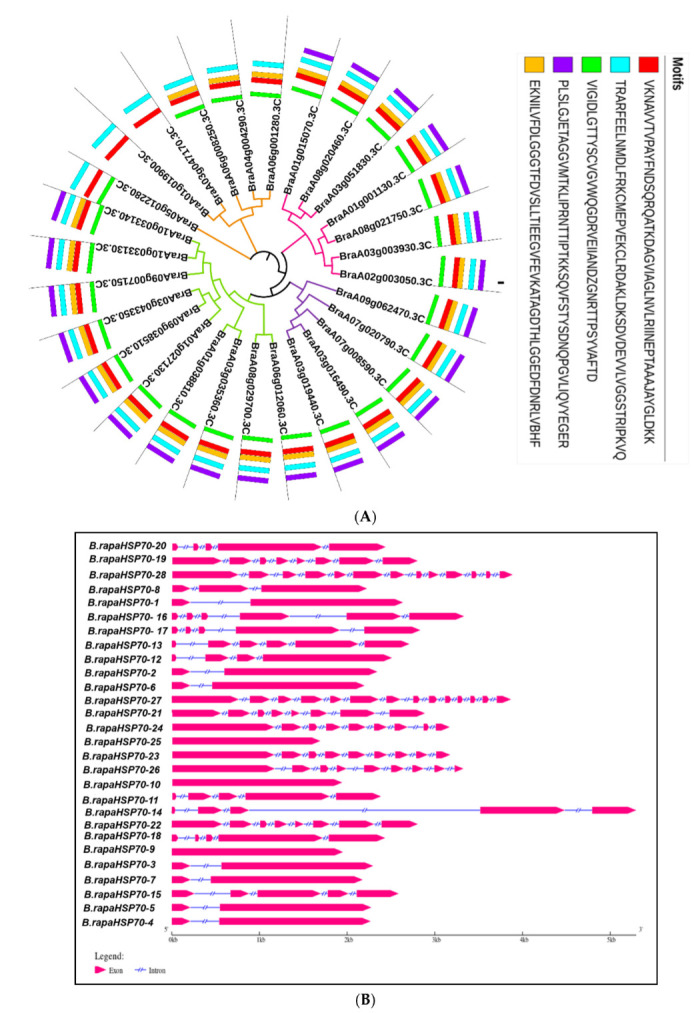
(**A**) Graphical representation of *B.rapaHSP70* conserved motif along with phylogenetic demonstration, divided into four groups, Group 1 (Brown), Group 2 (Pink), Group 3 (Purple) and Group 4 (Green). (**B**). Graphical representation of *B.rapaHSP70* gene structure (Intron, Exon).

**Figure 2:**
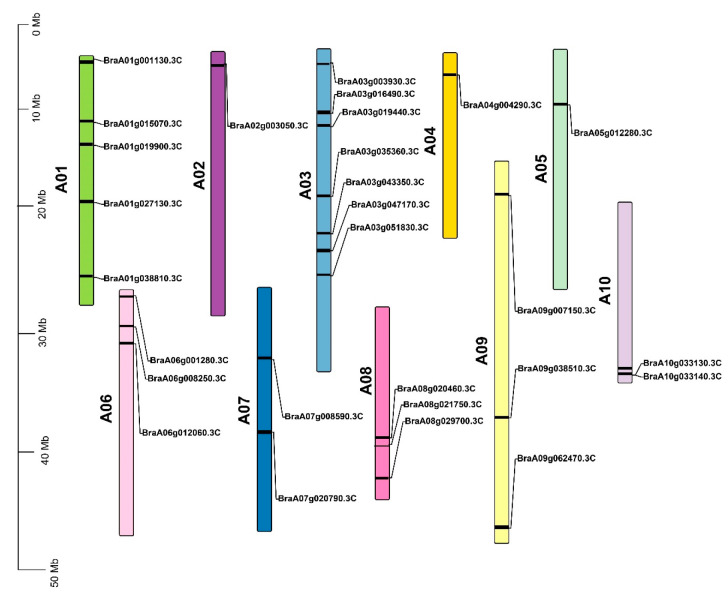
Chromosomal map of *B. rapa*, with distribution of HSP70 gene family members on *B. rapa* chromosomes by TBtools.

In this study, 28 genes were selected for further analysis using the A. thaliana query sequence (Gene I.D AT3G125801), and all selected genes were named *B.rapaHSP70-1*–*B.rapaHSP70-28*. These proteins were further analyzed for the HSP70 domain (**PF00012**) (Figure 1). Conserved motif along with phylogenetic tree demonstrate that all the *B.rapaHSP70s* are clustered into four groups (1, 2, 3 and 4), equal number and similar motif are present within each group except in group 4 (Figure 1A). Detailed information about the basic physical characteristics of the proteins is given in Table 1. Within the 28 HSP70 proteins, 12 members were localized in the cytoplasmic membrane, 5 in the endoplasmic reticulum, 4 in the chloroplast, 4 in the mitochondria, and 3 in the nucleus (Table 1). Gene structure predicts that the genes localized in the nucleus consists of the higher number of intron and exon while the cytoplasmic genes consist of the lower number of intron and exon (Figure 1B). The amino acid length of *B.rapaHSP70* proteins was 636–894 aa. Among these, the shortest protein was *B.rapaHSP70-15*(636 aa), and the longest was *B.rapaHSP70-28* (894 aa). The genes that translate into HSP70 proteins were distributed on 10 chromosomes, A01–A10. Among these, the maximum genes were distributed on chromosome A03 and the minimum on chromosomes A02, A04 and A05 (Figure 2).

### 3.2. Evolutionary Relationship of B.rapaHSP70 Gene

**Figure 3:**
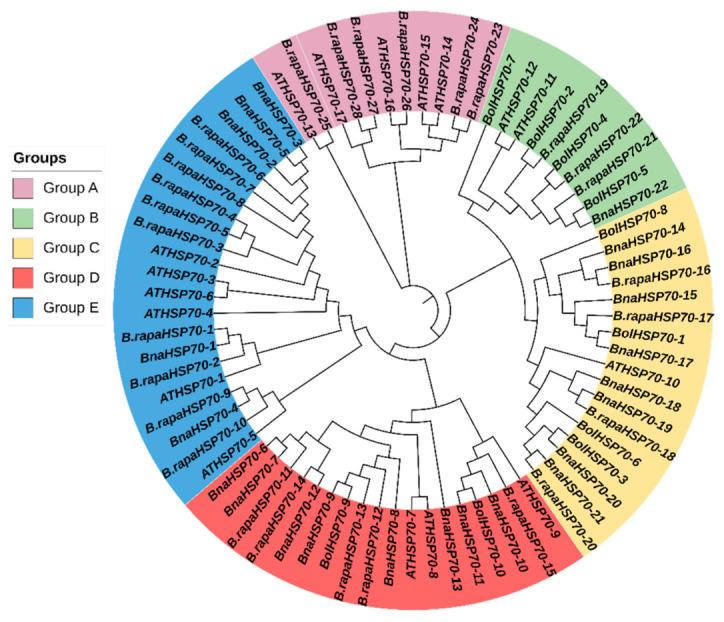
Comparative phylogenetic tree of the HSP70 proteins in *A*. *thaliana* (*AtHSP70*), *B*. *rapa* (*B.rapaHSP70*), *B*. *napus* (*BnaHSP70*), and *B*. *oleracea* (*BolHSP70*).

The evolutionary relationships between *B.rapaHSP70*, *B.nHSP70*, *B.olHSP70*, and *AtHSP70* were analyzed. Based on domains and a phylogenetic tree, 77 HSP70s were clustered into six major groups (A, B, C, D, and E) (Figure 3). The results from the evolutionary relationship showed that group A consists of 11 *B. rapa*, among which 5 members belonged to *Arabidopsis*. Group B was the largest group, containing 10 members, among which 4 members belonged to *B. rapa*, 1 member to *B. napus*, 3 to *B. oleracea*, and 2 to *Arabidopsis.* A total of 17 HSP70 members were clustered into group C, 4 from *B. rapa*, 8 from *B. napus*, 4 from *B. oleracea*, and 1 from *Arabidopsis*. Group D contained 18 members: 5 from *B. rapa*, 8 from *B. napus*, 2 from *B. oleracea*, and 3 from *Arabidopsis*. Group E consisted of 21 members: 10 from *B. rapa*, 5 from *B. napus*, and 6 from *Arabidopsis*. Hence, *B.rapaHSP70s* exhibited a close relationship with the other two Brassica crops (*B. oleracea* and *B. napus*) and *Arabidopsis*.

### 3.3. B.rapaHSP70 Gene Duplication and Ka/Ks Analysis

**Figure 4:**
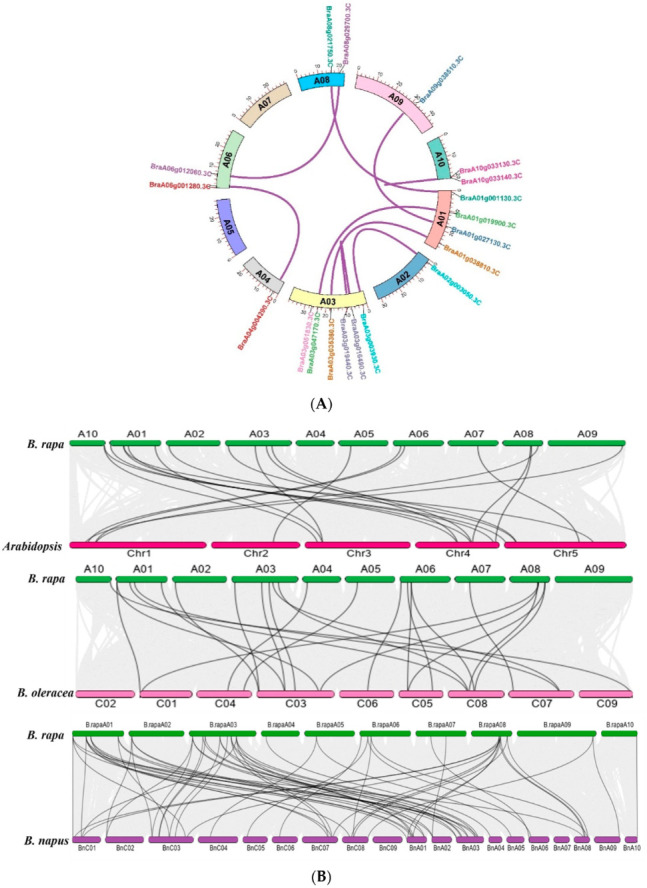
(**A**) Duplication of *B.rapaHSP70* genes. The lines indicate the association of segmentally duplicated pairs of genes. (**B**) Graphical illustration of syntenic association of *B*. *rapa* with *A*. *thaliana*, *B*. *oleracea*, and *B*. *napus*.

Gene duplication is one of the most important characteristics of a plant genomic structure, and it can occur by independent mechanisms resulting in segmental or tandem duplications. Due to the importance of gene duplications in the evolution of gene families in plants, we analyzed the gene duplication of putative *B.rapaHSP70* genes in the *B*. *rapa* genome. We detected duplicated gene couples among the 28 *B.rapaHSP70* genes identified in *B*. *rapa* (Figure 4A). Duplicated gene couples included BraA10g033140.3C/BraA10g033130.3C, BraA09g038510.3C/BraA01g027130.3C, BraA01g038810.3C/BraA03g035360.3C, BraA08g029700.3C/BraA06g012060.3C, BraA03g019440.3C/BraA03g016490.3C, BraA03g051830.3C/BraA08g020460.3C, BraA02g003050.3C/BraA03g003930.3C, BraA08g021750.3C/BraA01g001130.3C, BraA03g047170.3C/BraA01g019900.3C, and BraA06g001280.3C/BraA04g004290.3C. The value of Ka/Ks can be used as an indicator of the selection pressure of a gene during evolution. All of the values were <1, suggesting that the *B.rapaHSP70* genes were primarily evolved under the influence of purifying selection [26]. The divergence time million year ago (TMY) value was calculated based on T = Ks/2x, and the value of x was used according to [20] (Table 2).

### 3.4. B.rapaHSP70 Gene Duplication and Synteny Analysis of the B.rapaHSP70 Gene Family among Closely Related Species

BraA10g033140.3C/BraA10g033130.3C, BraA09g038510.3C/BraA01g027130.3C, BraA01g038810.3C/BraA03g035360.3C, BraA08g029700.3C/BraA06g012060.3C, BraA03g019440.3C/BraA03g016490.3C, BraA03g051830.3C/BraA08g020460.3C, BraA02g003050.3C/BraA03g003930.3C, BraA08g021750.3C/BraA01g001130.3C, BraA03g047170.3C/BraA01g019900.3C, and BraA06g001280.3C/BraA04g004290.3C were determined (Figure 4A), which shows that segmental duplication events occurred during evolution. Furthermore, a number of orthologs were identified among *A. thaliana*, *B. rapa* (AA), *B. napus* (AC), and *B. oleracea* (CC) using collinearity analysis. In addition, a comparative synteny analysis of HSP70 gene pairs among *B. rapa*, *B. napus*, *B. oleracea*, and *A. thaliana* was conducted (Figure 4B). We selected these species on the base of highest morphotype similarity of *B. rapa* (such as Chinese cabbage) and *B. oleracea* (Cabbage) and *B. napus* (Mustard) was included due the presence of both sub genome (A and C). *B.rapaHSP70* displayed the most collinearity with *B. napus*, followed by *A. thaliana* and *B. oleracea*. Results demonstrated that homologues genes from A genome of *B. rapa* are present in A as well as C genome of *B.napus*. Similar behavior was also observed in C genome of B. oleracea. These results suggest that, in addition to the whole genome duplication event, an independent duplication event also occurred during the evolution of these species.

### 3.5. Cis-Element Analysis in Promoter Regions of B.rapaHSP70 and Their Distribution

**Figure 5:**
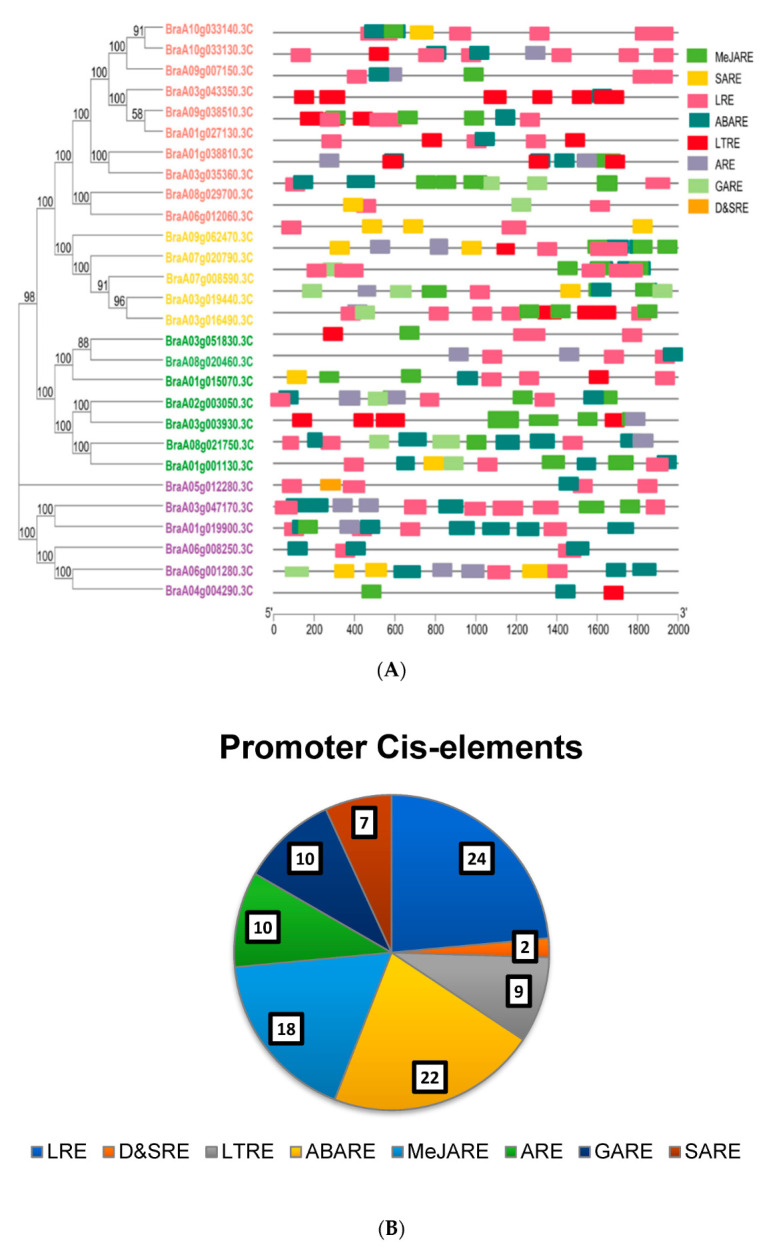
Graphical summary of identified cis-acting elements in HSP70 genes. (**A**) Phylogenetic tree with colored blocks of position and type of elements. (**B**) The pie chart shows the total identified elements and the number of each element in a specific color.

The sequence length of 2000 bp of all identified *B.rapaHSP70* genes was retrieved and uploaded to the PlantCare database to examine the cis-elements in the promoter region. The graphical representation (Figure 5A) illustrates a summary of the cis-acting elements. In total, 5 phytohormonal (ABA, abscisic acid; MeJA, methyl jasmonate, GA, gibberellin, and SA, salicylic acid) associated elements and 4 abiotic stress (temperature, drought, and light, and salinity) responsive elements were identified. Maximum elements were found for light response and auxin hormone response, and minimum elements were found for salicylic acid response in all promoters of the 28 *B.rapaHSP70* (Figure 5B and Table S1).

### 3.6. Prediction of the 3D Structures of B.rapaHSP70s

**Figure 6:**
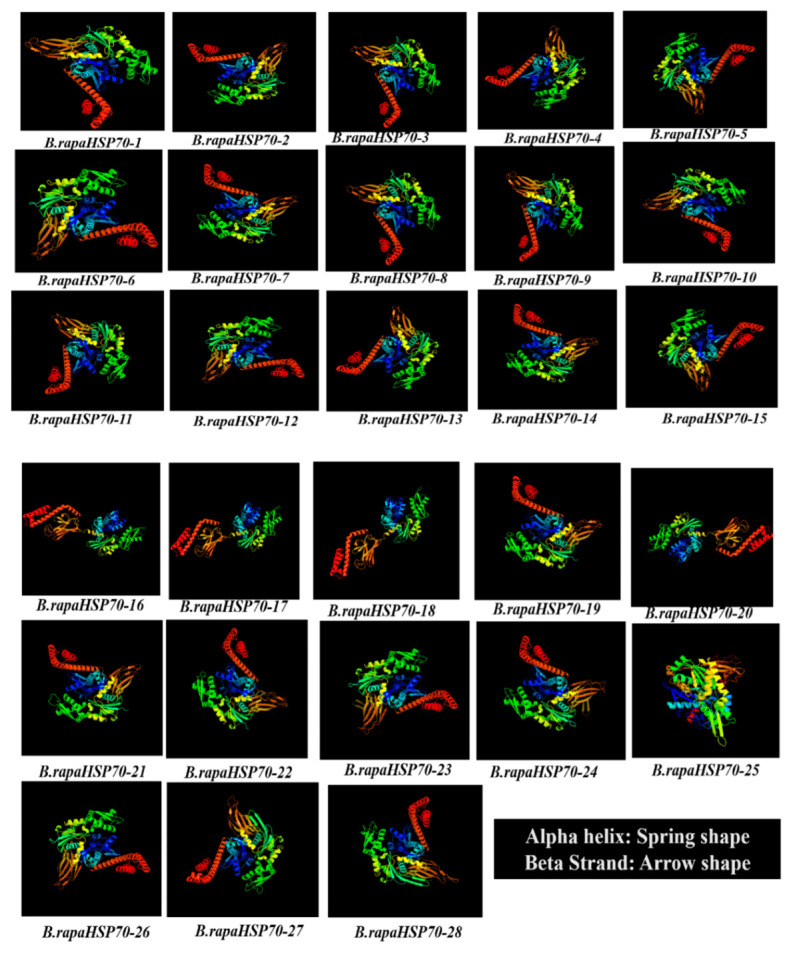
Predicted 3D models of *B.rapaHSP70* proteins. Models have been generated by using the Phyre 2 server in intensive mode. Models were visualized by rainbow colour from *N* to C terminus.

With the advent of graphical visualization of genomic data, 3D protein structures also explain the properties, as well as facilitate the comparative studies, of proteins. All 28 *B.rapaHSP70s* structures elucidate that all the HSP70 members in *B. rapa* possess the uniform monomer structure with alpha (α) helices and beta (β) sheets, because proteins with similar structures often have similar functions (Figure 6). Protein prediction also showed the existence of HSP70 domain (PF00012) in all the *B.rapaHSP70s*. The protein structures were modelled via c2khoA template at a confidence level of 100%. The overall fold of the *B.rapaHSP70* domain remains similar to other known HSP70 with the typical (αβα) topology, thus preserving conserved signature fold of HSP70 family with central mixed β-sheet sandwiched between α-helices [27]. On the bases of protein structure guide design, binding ligand can be determined, which are essential to bind with the external or internal residues. In addition, the tertiary structure of proteins predicted physical connections, distinct functional roles, independent divergence sequences, and conserved biological properties in the *B.rapaHSP70* protein family.

### 3.7. Genome-Wide Analysis of miRNA-Associated B.rapaHSP70 Genes

**Figure 7:**
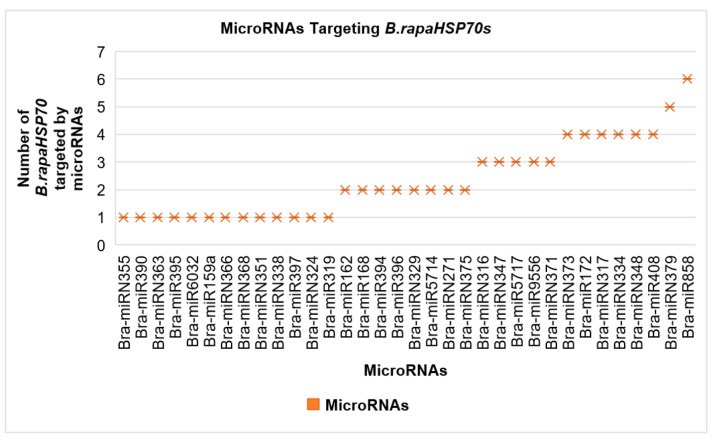
Smart graphical illustration of miRNAs targeting the HSP70 genes in *B. rapa*.

With all other factors, miRNAs have been reported to be crucial regulators of plant growth and development and have always been associated with stress responses in plants. Therefore, to further expand the knowledge of *B.rapaHSP70* genes, we identified the miRNAs associated with the identified genes (Figure 7). We found that 34 types of microRNA are associated with the *B.rapaHSP70s* to regulate their functioning. Such as Bra-miRN355, Bra-miR390, Bra-miRN363, Bra-miR395, Bra-miR6032, Bra-miR159a, Bra-miRN366, Bra-miRN368, Bra-miRN351, Bra-miRN338, Bra-miR397, Bra-miRN324, and Bra-miR319, all are targeting the only one *B.rapaHSP70* gene out of 28. Bra-miR162, Bra-miR168, Bra-miR394, Bra-miR396, Bra-miRN329, Bra-miR5714, Bra-miRN271, and Bra-miRN375 targeting the two different *B.rapaHSP70* genes. Bra-miRN316, Bra-miRN347, Bra-miR5717, Bra-miR9556, and Bra-miRN371 targeting the three *B.rapaHSP70* gene. Bra-miRN373, Bra-miR172, Bra-miRN317, Bra-miRN334, Bra-miRN348, and Bra-miR408 targeting the different four *B.rapaHSP70* gene. Bra-miRN379 targeting the five different *B.rapaHSP70s* and Bra-miR858 targeting the maximum, six different *B.rapaHSP70s* (Figure 7). Detailed information of *B.rapaHSP70s* along with their respective targeting microRNAs is summarized in Table 3. Raw information of the miRNA-targeted sites is presented in Table S2.

### 3.8. Functional Annotation Analysis of B.rapaHSP70 Genes

**Figure 8:**
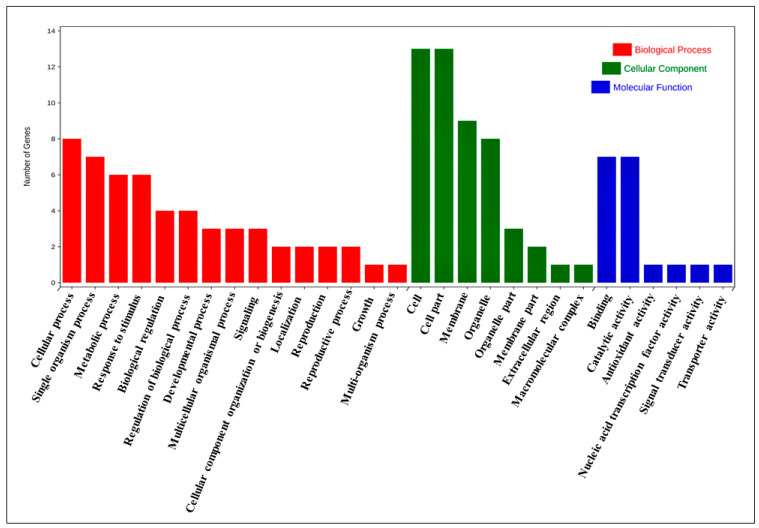
GO enrichment analysis of *B.rapaHSP70*.

Functional analysis using GO annotation and enrichment terms, such as molecular function (MF), cellular component (CC), and biological process (BP), were investigated to further explore the *B.rapaHSP70* genes’ functions. The annotation results for BP, MF, and CC exhibited several significantly enriched terms (Table S3). Some of the most enriched terms are highlighted in the subsequent section with a graphical illustration (Figure 8).

The GO-MF (Molecular Function) enrichment results detected 19 enriched terms, namely, protein serine/threonine phosphatase activity (GO:0004722 and GO:0004674), manganese ion binding (GO:0030145), pectate lyase activity (GO:0030570), 1-phosphatidylinositol binding (GO:0005545), glycoprotein binding (GO:0005515), DNA-binding transcription factor activity (GO:0003700), calmodulin binding (GO:0005516), cobalt ion binding (GO:0050897), clathrin binding (GO:0030276), ATP binding (GO:0005524 and GO:0005524), oligopeptide transmembrane transporter activity (GO:0015198), oxidoreductase activity (GO:0016491 and GO:0016491), glutathione peroxidase activity (GO:0004602), 2,3-bisphosphoglycerate-independent phosphoglycerate mutase activity (GO:0046537), protein serine/threonine kinase activity (GO:0004674), beta-amylase activity (GO:0016161), and maltose biosynthetic process (GO:0000024).

The GO-BP (Biological Process) enrichment results detected 26 enriched terms, namely, floral organ development (GO:0048437), oxidative stress responsive (GO:0006979), signal transduction (GO:0007165), signal transduction (GO:0006857), defense response (GO:0006952), pollen development (GO:0009555), male gamete generation (GO:0048235), signal transduction by cis-phosphorylation (GO:0007165), clathrin coat assembly (GO:0048268), regulation of transcription (GO:0006355), metabolic process (GO:0008152), salt stress responsive (GO:0009651), transmembrane transport (GO:0055085), plasma membrane organization (GO:0007009), glycolytic process (GO:0006096), abscisic acid responsive (GO:0009737), transmembrane receptor protein tyrosine kinase signaling pathway (GO:0007169), protein phosphorylation (GO:0006468), starch catabolic process (GO:0005983), cold stress responsive (GO:0009409), cadmium sensitivity/resistance (GO:0046686), regulation of meristem growth (GO:0010075), cadmium ion responsive (GO:0046686), temperature stimulus responsive (GO:0009409), maltose biosynthetic process (GO:0000024), and protein phosphorylation (GO:0006468).

The GO-CC (cellular component) enrichment results detected 29 enriched terms, including mitochondrial protein-transporting ATPase activity (GO:0005739 and GO:0005739), plasma membrane (GO:0005886), chloroplast envelope (GO:0009941), endomembrane system (GO:0012505), nucleus (GO:0005634 and GO:0005634), chloroplast (GO:0009507), apoplast (GO:0048046), endomembrane system (GO:0012505), chloroplast stroma (GO:0009570), cytosol (GO:0005829), clathrin coat (GO:0030118), mitochondrial envelope (GO:0005740), vacuole (GO:0005773), and integral component of membrane (GO:0016021) (Table S3).

### 3.9. Expression Profiling of B.rapaHSP70 Genes in Various Tissues

**Figure 9:**
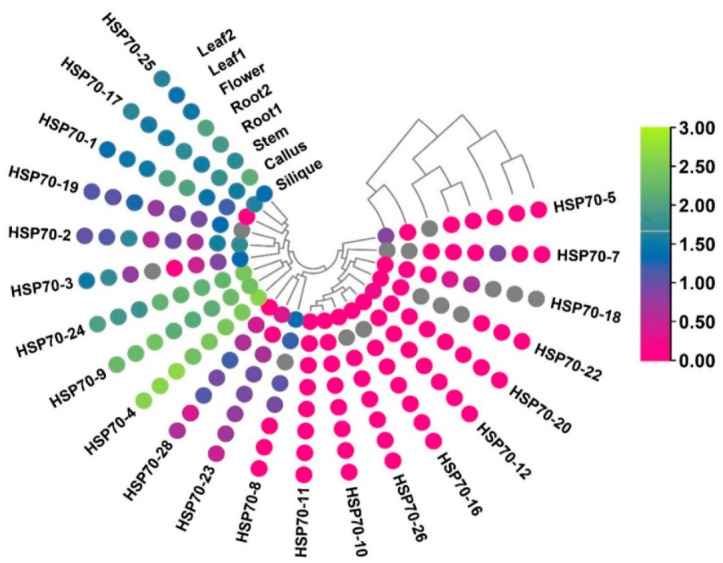
Expression pattern of HSP70 genes in different tissues based on fragments per kilobase of transcript per million mapped reads (FPKM). The color scheme represents expression intensity (purple, low expression; green, high expression) in different tissues.

To illustrate the transcript levels of the *B.rapaHSP70* genes, we examined 8 tissues and organs of *B. rapa* at various growth phases based on RNA-seq data of *B. rapa* (‘Chiifu-401-42’ variety; NCBI Gene Expression Omnibus; http://www.ncbi.nlm.nih.gov/geo/, accessed on 20 April 2022) under accession no. GSE43245. For instance, the expression patterns of most *B.rapaHSP70* genes in the silique, callus, and flower were higher than those of other tissues (Figure 9). Three *B.rapaHSP70* genes (*B.rapaHSP70-10*, *B.rapaHSP70-11*, *B.rapaHSP70-12*) showed no transcript changes in any tissue/organ.

The expression profiles of the *B.rapaHSP70* genes varied in various tissues and organs. In silique, the expression level of *B.rapaHSP70-4*, *B.rapaHSP70-9*, and *B.rapaHSP70-24* showing higher expression, and that of B.rapaHSP70-19 was lowest. In callus, *B.rapaHSP70-25* was highly expressed, and *B.rapaHSP70-10*, *B.rapaHSP70-11*, *B.rapaHSP70-12*, *B.rapaHSP70-16*, *B.rapaHSP70-18*, *B.rapaHSP70-20*, *B.rapaHSP70-4*, and *B.rapaHSP70-4* was the least expressed. In callus, *B.rapaHSP70-4, B.rapaHSP70-9, B.rapaHSP70-24* and *B.rapaHSP70-25* are expressing highly and *B.rapaHSP70-10, B.rapaHSP70-11, B.rapaHSP70-12, B.rapaHSP70-18, B.rapaHSP70-22* and *B.rapaHSP70-28* are showing no expression change. In stem, *B.rapaHSP70-4, B.rapaHSP70-9, B.rapaHSP70-24* and *B.rapaHSP70-25* are highly expressing, whereas *B.rapaHSP70-10*, *B.rapaHSP70-11*, *B.rapaHSP70-12*, *B.rapaHSP70-16*, *B.rapaHSP70-20*, *and B.rapaHSP70-26* showing no expression change. In roots, *B.rapaHSP70-4*, *B.rapaHSP70-9*, *B.rapaHSP70-24*, and *B.rapaHSP70-25* are highly expressed, whereas *B.rapaHSP70-10*, *B.rapaHSP70-11*, *B.rapaHSP70-12*, *B.rapaHSP70-16*, *B.rapaHSP70-20*, and *B.rapaHSP70-26* showing no expression change. In flower, *B.rapaHSP70-4*, *B.rapaHSP70-9*, and *B.rapaHSP70-24* are highly expressed genes, whereas *B.rapaHSP70-10*, *B.rapaHSP70-11*, *B.rapaHSP70-12*, *B.rapaHSP70-16*, and *B.rapaHSP70-22* showing zero expression change. Lastly, the expression was analyzed in leaf, according to the expression pattern the *B.rapaHSP70-4*, *B.rapaHSP70-9*, *B.rapaHSP70-17*, *B.rapaHSP70-24*, and *B.rapaHSP70-25* showing higher expression whereas *B.rapaHSP70-10*, *B.rapaHSP70-11*, *B.rapaHSP70-12*, *B.rapaHSP70-16*, *B.rapaHSP70-20*, and *B.rapaHSP70-26* showing no expression change. These findings suggest that these candidate genes may play diverse roles in regulating *B. rapa* growth processes (Figure 9).

### 3.10. B.rapaHSP70 Gene Expression in Response to Heat and Cold Stress

To examine the response of *B.rapaHSP70s* under cold and heat stress, we investigated the gene expression of *B.rapaHSP70s* in Chinese cabbage plants grown at 4 °C under cold stress and 45 °C under heat stress conditions. qRT-PCR was used to investigate the transcript levels of each *B.rapaHsp70* gene with three biological repetitions and two technical repetitions. The primers used in this experiment are provided in Table S4. Generally, the relative expression level of the *B.rapaHSP70* genes under all stress conditions fluctuated during the 24-h treatments.

**Figure 10:**
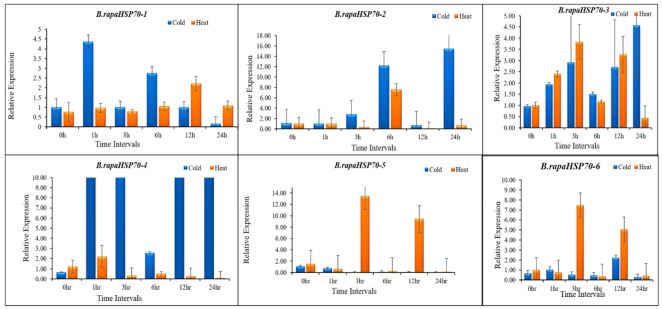

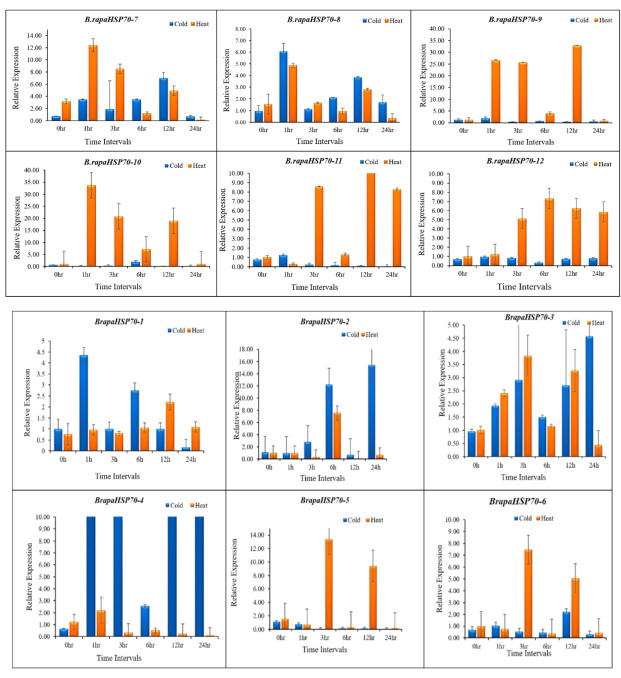

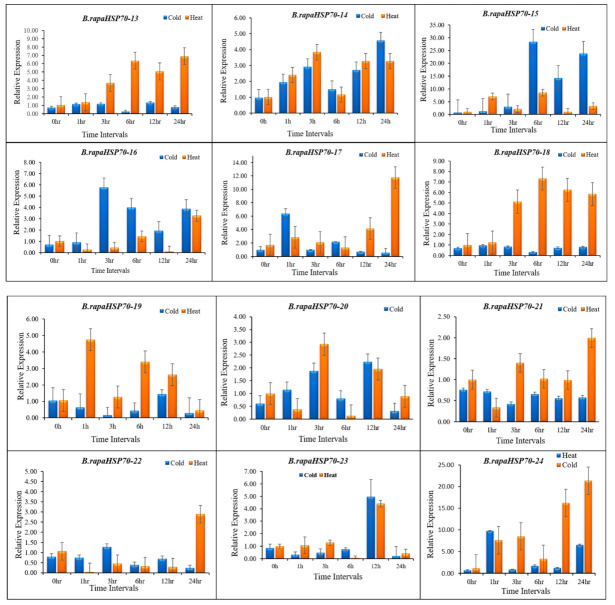

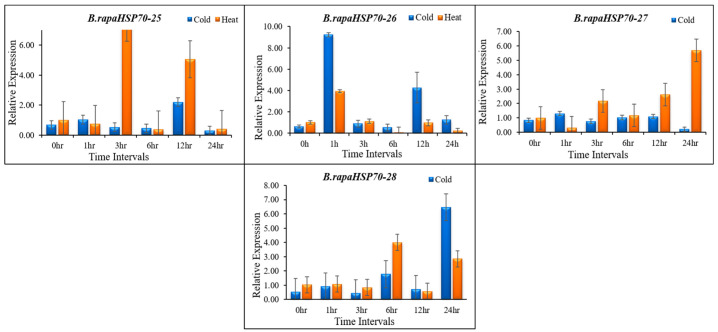
*B.rapaHSP70* gene expression in Chinese cabbage leaves after exposure to cold and heat stress. Heat treatment was represented with orange bars, and cold treatment with blue bars. Samples were taken at 0, 1, 3, 6, 12, and 24 h after heat stress treatment.

Most of the *B.rapaHSP70* genes were sensitive to heat stress, and most were highly regulated under high-temperature stress compared to cold stress. A few *B.rapaHSP70s* showed no significant expression differences after being treated under different time intervals. The expression levels of 16 *B.rapaHSP70s* (*B.rapaHSP70-1*, *-2*, *-3*, *-4*, *-8*, *-14*, *-15*, *-16*, *-19*, *-20*, *-22*, *-23*, *-24*, *-25*, *-27*, and *-28*) were upregulated under high-temperature stress at different time intervals, and most were highly expressed after 24 h of heat stress. The expression levels of 16 *B.rapaHSP70s* (*B.rapaHSP70-1*, *-2*, *-3*, *-4*, *-7*, *-8*, *-13*, *-14*, *-17*, *-20*, *-21*, *-22*, *-23*, *-24*, *-26*, and *-28*) were high at different time intervals under cold stress (Figure 10 and Figure S5). qRT-PCR analysis showed that most of the *B.rapaHSP70*s expression were higher under heat stress as compared to cold stress. Interestingly, it is noticeable that *B.rapaHSP70-3*, *B.rapaHSP70-14*, and *B.rapaHSP70-20* showed higher expression under both stress conditions. With the comparison of digital expression pattern results and our experimental analysis, there is significant difference in the expression of *B.rapaHSP70* genes. The *B.rapaHSP70* genes that are showing no expression changes in the normal condition such as *B.rapaHSP70-10*, *B.rapaHSP70-11*, *B.rapaHSP70-12*, and *B.rapaHSP70-16*, there expression is higher under the heat and cold shock conditions. All 28 *B.rapaHSP70s* exhibited a change in expression under stress conditions, these findings may help the breeders to improve the heat and cold tolerance in the Chinese cabbage.

## 4. Discussion

In this cutting-edge era of modern science, with the availability of open-source databases of whole genome sequences, HSP70 gene families have been explored in model plants and a diverse range of horticultural plant species, such as soybean. With the availabilities of the whole genome sequence of many plants, several HSP70 families have been identified, such as in *A. thaliana* and *N. tabacum*, as well as in different horticultural plants, such as *B. napus*, *B. oleracea*, and pumpkin [28,29,30,31,32]. However, this technique (genome-wide analysis) has not yet been employed in the HSP70 family in *Brassica rapa*.

### 4.1. Characterization of the B.rapaHSP70 Gene Family in B. rapa

The model plant *A. thaliana* belongs to the same family (Brassicaceae) as *B. rapa*. We identified 28 gene family members of HSP70 in *B. rapa*, which is higher than that found in *Arabidopsis* (11 AtHSP70) [8]. The identified genes were analyzed to explore their physical properties. There was no difference in the location of exon/intron boundaries or coding sequences. However, there were significant variances in the sequences and sizes of the introns in each of the 28 HSP70 genes.

Notably, genes within the same phylogenetic subgroup had similar motif compositions and exon/intron structures (Figure 1 and Table 1). Genes without introns or those with few introns have been found to have enhanced plant expression levels in some studies [33]. In addition, the gene classifications of *B.rapaHSP70* were confirmed by the correlation between intron numbers and motif arrangements in combination with phylogeny. A phylogenetic tree was created to show the evolutionary relationships between *B. rapa*, *B. napus*, *B. oleracea*, and Arabidopsis. Recently, research has examined the evolutionary associations among these species [32,34].

During the genome evolution process, gene duplications and chromosomal segments are major forces in the evolution of plant genome structure and content [35]. Tandem or segmental duplication events in which two or more neighboring genes duplicate on the same chromosome or on different chromosomes, respectively, can occur [36]. Based on the importance of duplication events, gene duplication events were explored to further understand the expansion mechanism of the *B.rapaHSP70* family genes and we find all the duplicated *B.rapaHSP70s* are segmental duplicated.

### 4.2. Promoter Analysis and Gene Ontology

Previously, a total of 11 HSP70 genes from *A. thaliana* were identified and subjected to promoter analysis to investigate the presence of temperature-associated (heat shock element (HSE)) and dehydration-associated cis-elements (dehydration responsive element; DRE) [8]. Based on our findings, most HSP70 genes were predicted to be light responsive, presenting their effects when light or heat intensities were altered.

These stress-related cis elements revealed the involvement of HSP70 genes in *B. rapa* stress tolerance. Furthermore, GO enrichment analysis supports the findings of the HSP70 gene association with stress tolerance (Table S2). The molecular mechanism and functional investigation of stress-related HSP70 genes in Brassica crops should be a focus in the future to understand the underlying mechanisms.

### 4.3. miRNA: Key Players in the Regulation of Stress Responses

Several studies have revealed the importance and role of microRNAs (miRNAs) in post-transcriptional gene regulation [37]. In the past few years, numerous miRNAs have been identified in *B. rapa* that respond to different environmental stress conditions [13,38]. For instance, miR166 has been upregulate due to sunlight in maize crops and under heat stress in Chinese cabbage [39]. Similarly, miR393 has been reported to be associated with cold stress in tea plants (*Camellia sinensis*) [40]. These studies suggest that these Brassica-associated miRNAs might play decisive roles against numerous stressors by modifying the transcript levels of HSP70 genes. In our study, we found that Bra-miR162, Bra-miR165, Bra-miR172, Bra-miR316, Bra-miR375, Bra-miR396, Bra-miR397, Bra-miR408, Bra-miR858, Bra-miR5714, and Bra-miR5717 families targeted the *B.rapaHSP70* genes (Table 3 and Table S3).

## 5. Differential Expression Analysis of *B.rapaHSP70* Genes in Chinese Cabbage Leaves under Heat and Cold Stress

The expression patterns of genes are associated with their function [41]. Several studies have reported the use of qRT-PCR for investigating transcript levels, such as expression profiling of *StHSP20* under heat stress. In another study, the results verified the involvement of *B.rapaHSP70* proteins in thermotolerance [42]. Until now, few HSP70 genes have been identified and functionally described in *B. rapa* and other plant species. In this study, we laid a foundation for recognizing signaling controlled by HSP70 proteins under biotic and abiotic stress conditions.

## 6. Conclusions

In the current study, we performed a genome-wide analysis of the HSP70 gene family in *B. rapa*, and 28 putative members were identified. In silico analyses were performed, including gene structure, distribution, phylogenetic relationship, and syntenic studies, which helped to explore the evolutionary properties of the HSP70 gene family in *B. rapa*. In addition, targeted miRNAs, promotor cis-acting regulatory elements, and gene ontology (GO) were executed. The finding of all these analysis elucidating that *B.rapaHSP70s* are targeted by 34 different families of microRNAs, these genes are highly responsive to light, temperature and phytohormones, and *B.rapaHSP70s* are involved in the Biological, cellular and molecular functioning of *B. rapa,* respectively. Three dimensional protein structural knowledge can help improve crop properties, such as improving stress resistance and biomass yield. Furthermore, quantitative real-time PCR results indicated that HSP70 genes are strongly involved in heat and cold stress responses in *B. rapa* plants. To a large extent, all analyses performed on the HSP70 gene family will lay the foundation for further studies of molecular and physiological functions in Brassica crops.

## Figures and Tables

**Table 1:** *B.rapaHSP70s* gene information retrieved for genome wide analysis.

Transcript ID	Gene Name	Chr Position	Location Start–End	Strand	CDS(BP)	Instability Index	Protein Length (A.A)	Protein MW (KDa)	PI	GRAVY	Intron, Exon	Sub-Cellular Localization
BraA01g038810.3C	*B.rapaHSP70-1*	A01	26269513–26272150	Positive	1953	32.26	650	71.27	5.13	−0.429	1,2	Cytoplasm
BraA03g035360.3C	*B.rapaHSP70-2*	A03	17438369–17440713	Negative	1958	33.17	652	71.38	5.13	−0.443	1,2	Cytoplasm
BraA09g007150.3C	*B.rapaHSP70-3*	A09	4091084–4093380	Negative	1944	36.12	647	70.94	5.04	−0.401	1,2	Cytoplasm
BraA10g033140.3C	*B.rapaHSP70-4*	A10	20445814–20448082	positive	1941	36.2	646	70.87	5.07	−0.396	1,2	Cytoplasm
BraA10g033130.3C	*B.rapaHSP70-5*	A10	20434824–20437099	Negative	1941	36.2	646	70.87	5.07	−0.396	1,2	Cytoplasm
BraA03g043350.3C	*B.rapaHSP70-6*	A03	21842760–21844958	Positive	1953	34.3	650	71.18	5.07	−0.419	1,2	Cytoplasm
BraA09g038510.3C	*B.rapaHSP70-7*	A09	30333133–30335310	Positive	1950	35.5	649	71.05	5.02	−0.416	1,2	Cytoplasm
BraA01g027130.3C	*B.rapaHSP70-8*	A01	17439040–17441266	Negative	1986	36.31	661	72.34	5.11	−0.407	2,3	Cytoplasm
BraA08g029700.3C	*B.rapaHSP70-9*	A08	20365638–20367590	Positive	1953	35.47	650	71.34	5.28	−0.434	0,1	Cytoplasm
BraA06g012060.3C	*B.rapaHSP70-10*	A06	6488327–6490270	Negative	1944	35.51	647	71.04	5.48	−0.408	0,1	Cytoplasm
BraA07g008590.3C	*B.rapaHSP70-11*	A07	8612041–8614425	Negative	2010	30	669	73.84	5.08	−0.477	4,5	Endoplasmic Reticulum
BraA03g019440.3C	*B.rapaHSP70-12*	A03	9196468–9198979	Positive	2010	27.96	669	73.62	5.08	−0.464	3,4	Endoplasmic Reticulum
BraA03g016490.3C	*B.rapaHSP70-13*	A03	7627325–7630035	Negative	2010	27.79	669	73.63	5.08	−0.468	5,6	Endoplasmic Reticulum
BraA07g020790.3C	*B.rapaHSP70-14*	A07	17199507–17204802	Negative	1998	30.99	665	73.49	5.11	−0.466	4,5	Endoplasmic Reticulum
BraA09g062470.3C	*B.rapaHSP70-15*	A09	43345497–43348084	Positive	1911	32.8	636	70.55	4.94	−0.48	4,5	Endoplasmic Reticulum
BraA02g003050.3C	*B.rapaHSP70-16*	A02	1492088–1495420	Positive	2043	34.42	680	72.87	5.82	−0.335	5,6	Mitochondrial
BraA03g003930.3C	*B.rapaHSP70-17*	A03	1680387–1683221	Positive	2046	33.94	681	72.67	5.55	−0.336	4,5	Mitochondrial
BraA08g021750.3C	*B.rapaHSP70-18*	A08	16460538–16462970	Negative	2031	36.79	676	72.48	5.79	−0.281	4,5	Mitochondrial
BraA01g015070.3C	*B.rapaHSP70-19*	A01	7988766–7991570	Positive	2136	31.48	711	76.1	5.09	−0.316	4,5	Chloroplast
BraA01g001130.3C	*B.rapaHSP70-20*	A01	523816–526255	Positive	2049	38.26	682	73.01	5.56	−0.306	7,8	Mitochondrial
BraA03g051830.3C	*B.rapaHSP70-21*	A03	26797918–26800806	Positive	2133	27.71	710	75.91	5.27	−0.316	7,8	Chloroplast
BraA08g020460.3C	*B.rapaHSP70-22*	A08	15677635-15680441	Negative	2154	28.52	717	76.42	5.14	−0.313	7,8	Chloroplast
BraA06g001280.3C	*B.rapaHSP70-23*	A06	802745–805920	Positive	2457	39.54	818	90.29	5.17	−0.402	8,9	Cytoplasmic
BraA04g004290.3C	*B.rapaHSP70-24*	A04	2645163–2648333	Negative	2373	40.91	790	87.16	5.23	−0.4	8,9	Cytoplasmic
BraA05g012280.3C	*B.rapaHSP70-25*	A05	6664594–6666288	Negative	1695	41.55	564	60.81	5.39	0.051	0,1	Chloroplast
BraA06g008250.3C	*B.rapaHSP70-26*	A06	4497297–4500623	Positive	2286	47.68	761	84.54	5.73	−0.493	8,9	Nuclear
BraA03g047170.3C	*B.rapaHSP70-27*	A03	23836812-23840680	Positive	2631	42.89	876	97.59	5.78	−0.483	13,14	Nuclear
BraA01g019900.3C	*B.rapaHSP70-28*	A01	10687460–10691347	Negative	2685	44.47	894	99.97	5.89	−0.499	12,13	Nuclear

**Table 2:** Ka/Ks values of *B.rapaHSP70* duplicated genes.

Seq_1	Seq_2	Ka	Ks	Ka_Ks	T(MYA)
BraA10g033140.3C	BraA10g033130.3C	0	0.0983403	0	3278010.814
BraA09g038510.3C	BraA01g027130.3C	0.0056856	0.2035601	0.027931049	6.785335295
BraA01g038810.3C	BraA03g035360.3C	0.0086499	0.9733226	0.008887026	32.44408789
BraA08g029700.3C	BraA06g012060.3C	0.0273781	0.7299868	0.037504912	24.33289231
BraA03g019440.3C	BraA03g016490.3C	0.0035436	0.2455705	0.014430195	8.185684806
BraA03g051830.3C	BraA08g020460.3C	0.0218032	0.5165388	0.042210144	17.21796007
BraA02g003050.3C	BraA03g003930.3C	0.0206864	0.3771192	0.054853846	12.57063953
BraA08g021750.3C	BraA01g001130.3C	0.0253008	0.4383311	0.057720824	14.61103702
BraA03g047170.3C	BraA01g019900.3C	0.0506028	0.315182	0.160551192	10.50606656
BraA06g001280.3C	BraA04g004290.3C	0.0160146	0.3383936	0.047325361	11.27978551

**Table 3:** MicroRNA targeting *B.rapaHSP70s*.

MicroRNAs	MicroRNA Targeting *B.rapaHSP70s*					
Bra-miRN355	*B.rapaHSP70-3*					
Bra-miR390	*B.rapaHSP70-8*					
Bra-miRN363	*B.rapaHSP70-10*					
Bra-miR395	*B.rapaHSP70-13*					
Bra-miR6032	*B.rapaHSP70-14*					
Bra-miR159a	*B.rapaHSP70-15*					
Bra-miRN366	*B.rapaHSP70-15*					
Bra-miRN368	*B.rapaHSP70-15*					
Bra-miRN351	*B.rapaHSP70-16*					
Bra-miRN338	*B.rapaHSP70-19*					
Bra-miR397	*B.rapaHSP70-23*					
Bra-miRN324	*B.rapaHSP70-25*					
Bra-miR319	*B.rapaHSP70-26*					
Bra-miR162	*B.rapaHSP70-21*	*B.rapaHSP70-22*				
Bra-miR168	*B.rapaHSP70-2*	*B.rapaHSP70-13*				
Bra-miR394	*B.rapaHSP70-14*	*B.rapaHSP70-27*				
Bra-miR396	*B.rapaHSP70-23*	*B.rapaHSP70-24*				
Bra-miRN329	*B.rapaHSP70-4*	*B.rapaHSP70-5*				
Bra-miR5714	*B.rapaHSP70-18*	*B.rapaHSP70-22*				
Bra-miRN271	*B.rapaHSP70-16*	*B.rapaHSP70-17*				
Bra-miRN375	*B.rapaHSP70-21*	*B.rapaHSP70-23*				
Bra-miRN316	*B.rapaHSP70-15*	*B.rapaHSP70-23*	*B.rapaHSP70-24*			
Bra-miRN347	*B.rapaHSP70-6*	*B.rapaHSP70-12*	*B.rapaHSP70-13*			
Bra-miR5717	*B.rapaHSP70-20*	*B.rapaHSP70-21*	*B.rapaHSP70-22*			
Bra-miR9556	*B.rapaHSP70-9*	*B.rapaHSP70-13*	*B.rapaHSP70-15*			
Bra-miRN371	*B.rapaHSP70-19*	*B.rapaHSP70-21*	*B.rapaHSP70-22*			
Bra-miRN373	*B.rapaHSP70-1*	*B.rapaHSP70-22*	*B.rapaHSP70-27*	*B.rapaHSP70-28*		
Bra-miR172	*B.rapaHSP70-2*	*B.rapaHSP70-9*	*B.rapaHSP70-14*	*B.rapaHSP70-23*		
Bra-miRN317	*B.rapaHSP70-15*	*B.rapaHSP70-19*	*B.rapaHSP70-27*	*B.rapaHSP70-28*		
Bra-miRN334	*B.rapaHSP70-7*	*B.rapaHSP70-8*	*B.rapaHSP70-16*	*B.rapaHSP70-17*		
Bra-miRN348	*B.rapaHSP70-3*	*B.rapaHSP70-4*	*B.rapaHSP70-5*	*B.rapaHSP70-8*		
Bra-miR408	*B.rapaHSP70-6*	*B.rapaHSP70-11*	*B.rapaHSP70-21*	*B.rapaHSP70-22*		
Bra-miRN379	*B.rapaHSP70-1*	*B.rapaHSP70-2*	*B.rapaHSP70-25*	*B.rapaHSP70-27*	*B.rapaHSP70-28*	
Bra-miR858	*B.rapaHSP70-12*	*B.rapaHSP70-13*	*B.rapaHSP70-18*	*B.rapaHSP70-19*	*B.rapaHSP70-20*	*B.rapaHSP70-22*

## Data Availability

The datasets used and/or analyzed during the current study are available from the corresponding author on reasonable request. However, most of the data is shown in Supplementary files.

## Supplementary Materials

The following supporting information can be downloaded at: https://www.mdpi.com/article/10.3390/cells11152316/s1, Table S1; Cis-Element Analysis of *B.rapaHSP70* Gene Promoters. Putative cis-elements of the 28 *B.rapaHSP70* genes identified via PlantCARE web-based tool, Table S2; Genome-Wide Analysis of miRNA-Associated *B.rapaHSP70* Genes. Detailed information of the miRNA-targeted sites determined by psRNATarget database, Table S3; Functional Annotation Analysis of *B.rapaHSP70* Genes. Detailed information of the GO annotation data retrived from Brassica database, Table S4; Primers used in qRT-PCR. Expression patteren analysis in Response to Heat and Cold Stress, Table S5; Average fold expression and standard error values of *B.rapaHSP70s*. Relative expression level of the *B.rapaHSP70* genes under heat and cold Stress.
